# Association between bacterial resistance profile and the development of intra-abdominal abscesses in pediatric patients with perforated appendicitis: cohort study

**DOI:** 10.1007/s00383-023-05570-3

**Published:** 2023-12-11

**Authors:** Luz Nélida Garzon-González, Laura Tatiana Padilla, Felipe Patiño, María Alejandra Hernández, Juan Valero, Iván Dario Molina, Fernando Fierro Ávila, German Camacho-Moreno

**Affiliations:** 1 Paediatric Surgery Resident, Department of Surgery, Faculty of Medicine, Universidad Nacional de Colombia, Bogotá, Colombia; 2 HOMI-Fundación Hospital Pediátrico La Misericordia, Cra 14 # 1-65, Bogotá, Colombia; 3 Institute of Clinical Research, Universidad Nacional de Colombia, Bogotá, Colombia; 4 Department of Surgery, Faculty of Medicine, HOMI-Fundación Hospital Pediátrico La Misericordia, Universidad Nacional de Colombia, Bogotá, Colombia; 5 Department of Paediatrics, Faculty of Medicine, HOMI-Fundación Hospital Pediátrico La Misericordia, Universidad Nacional de Colombia, Bogotá, Colombia

**Keywords:** Intra-abdominal abscess, Perforated appendicitis, Antimicrobial drug resistance, Children, Risk factors

## Abstract

**Purpose:**

The objective of this study was to determine the association between the presence of a microorganism resistant to the antibiotic used in empirical therapy and the development of intra-abdominal abscesses in children with perforated appendicitis.

**Methods:**

A prospective cohort study was conducted in patients under 18 years of age who underwent laparoscopic appendectomy between November 1, 2019, and September 30, 2020, in whom perforated appendicitis was documented intraoperatively. Peritoneal fluid samples were taken for bacteria culture purposes, and clinical and microbiological data were collected from all patients.

**Results:**

A total of 232 patients were included in the study. The most isolated microorganisms were *Escherichia coli *(*80.14%*) and *Pseudomonas aeruginosa* (7.45%). In addition, 5.31% of *E. coli* isolates were classified as ESBL-producing organisms. No association was found between a germ resistant to empiric antimicrobial therapy and the development of a postoperative intra-abdominal abscess. Multivariate analysis showed that being a high-risk patient on admission (OR 2.89 (*p* = 0.01)) was associated with the development of intra-abdominal abscesses postoperatively.

**Conclusion:**

*E. coli* was the most commonly isolated microorganism, with a low rate of ESBL-producing isolates. No association between resistance and risk of postoperative intra-abdominal abscess was found. However, it was identified that being a high-risk patient on admission was associated with this complication.

**Type of study:**

Prognosis study.

**Level of evidence:**

Level I.

## Introduction

Abdominal pain accounts for 7–10% of all visits to emergency departments, being acute appendicitis the most common cause of abdominal pain in the right lower quadrant [1]. Acute appendicitis is the most frequent reason for surgery in the pediatric population, with a peak incidence between the ages of 10 and 19 years [2]. Appendicitis is classified into two types: perforated and non-perforated. Perforated appendicitis occurs when there is a visible hole in the appendix or when a free fecalith is found in the peritoneal cavity [3, 4].

Even though the most commonly isolated pathogens in patients with acute appendicitis are *Enterobacteriaceae* (*Escherichia coli, Klebsiella* sp), Gram-positive cocci (*Enterococcus* sp), and strict anaerobic microorganisms (*Bacteroides fragilis*) [5, 6], the susceptibility of bacteria to antibiotics can vary from one hospital to another.

Intra-abdominal abscesses have been described as a complication of appendicitis in 3.7% to 20% of cases, and it has also been reported that they increase morbidity [2–8]. The association between having a microorganism resistant to empiric antibiotic therapy and the development of complications such as intra-abdominal abscesses has not been studied in depth. Furthermore, local antimicrobial resistance profiles of isolated bacteria have been scarcely addressed in low- and middle-income countries. An empirical antimicrobial therapy based on local antibiotic resistance profiles is used for the management of acute appendicitis, thus it can be inferred that the presence of antibiotic resistant germs is a risk factor for the development of intra-abdominal abscesses.

The main objective of this study was to determine the association between the presence of a microorganism resistant to the empiric antibiotic treatment used and the development of intra-abdominal abscesses in patients with acute perforated appendicitis who underwent surgery at HOMI, *Fundación Hospital Pediátrico La Misericordia*. The secondary objective was to describe the local antibiotic susceptibility and resistance profiles.

## Methods

A prospective cohort study was conducted in patients under 18 years of age who underwent laparoscopic appendectomy at HOMI, *Fundación Hospital Pediátrico La Misericordia*, a tertiary care referral hospital specialized in the care of children in Colombia, where 1 081 to 1 626 appendectomies are performed every year. The study included all patients in whom perforated appendicitis was documented intraoperatively, as well as the presence of at least one germ in peritoneal fluid between November 1, 2019, and September 30, 2020. Patients with plastron appendicitis that were initially treated with antibiotics were excluded. This study was approved by the ethics committee of the *HOMI—Fundación Hospital Pediátrico La Misericordia*, as stated in Minutes No. 0.12 CEI 19-18, and an informed consent form was signed by the parents or legal guardians of each participant. The Declaration of Helsinki was also considered [9].

Sociodemographic data and data regarding symptom duration and treatments received prior to hospital admission were collected from all participants. During surgery, a peritoneal fluid aspirate was taken using a suction laparoscopy cannula and distributed into 3 bottles: 1 aerobic blood culture bottle, 1 anaerobic blood culture bottle, and 1 dry tube [10]. Information on the surgery, hospitalization, and discharge was also collected, as well as data obtained during the follow-up visit, which took place at least 15 days after the surgery. Patients who could not attend the follow-up visit were contacted by electronic means. During the SARS-CoV-2 pandemic quarantine, patients were contacted by telephone. Information was collected prospectively and stored in Excel, and then processed using STATA 14 software, version 14.

At HOMI, appendectomies are always performed by a pediatric surgery resident under the supervision of a pediatric surgeon, except in complex cases, where the pediatric surgeon is the lead surgeon. All appendectomies are performed laparoscopically unless the clinical condition of the patient or abdominal distension contraindicate this approach. During laparoscopy, the caecal appendix is removed, pus is aspirated from the abdominal cavity, and purulent membranes are removed. Routine irrigation of the peritoneal cavity is not performed. All patients receive prophylaxis with ampicillin/sulbactam 200 mg/kg/d. If a patient with a high risk of abscesses or presenting generalized peritonitis (peritonitis in 3 or more quadrants) is detected, treatment is switched to piperacillin/tazobactam (300 mg/kg/d) in the immediate postoperative period. Based on the Surviving Sepsis Campaign International Guidelines for children [11], the presence of septic shock, previous use of antibiotics, and immunodeficiency (due to concomitant disease or medication) are considered high-risk factors in the present study.

Patients who do not have a fever on the third postoperative day, have an adequate tolerance to a normal diet and have adequate pain control are candidates for oral antibiotic treatment on an outpatient basis. This decision is made based on the follow-up complete blood count results and the results of the C-reactive protein (CRP) test performed on the same day. The antibiotic is administered on the day of discharge to complete the proposed regimen. The other patients continue to receive intravenous antibiotic treatment until discharge.

The culture report of the sample taken during the procedure is usually obtained at 72 h. If the report shows a microorganism resistant to the antibiotic that the patient is receiving, but the patient is asymptomatic and the blood count does not show leukocytosis or neutrophilia, the antibiotic therapy is not modified.

### Funding data

The funding for the processing of the laboratory samples was provided by HOMI—*Fundación Hospital Pediátrico La Misericordia.* The authors received no monetary compensation for this study.

## Results

### Univariate analysis

Between November 1, 2019 and September 30, 2020, 1067 children underwent appendectomy at HOMI. Of these, 408 (38.23%) had perforated appendicitis. An adequate peritoneal fluid sample for bacteria culture purposes was not possible in 152 patients, so they were excluded from the study. A total of 256 samples were collected for culture, and 21 patients were excluded because their culture was negative, 2 because their pathology report did not show acute appendicitis, and 1 because informed consent was not available. Data from the remaining 232 patients were collected.

The average age of the patients was 10.10 years (SD 3.74) and 55.6% were males. At admission, all patients had abdominal pain, followed by vomiting (90.52%), anorexia (79.31%), nausea (76.29%), and diarrhea (41.81%). The median time elapsed between symptom onset and consultation was 41.06 h (Table 1).

**Table 1:** Sociodemographic data of the patients

Basic characteristics		Patients, *n* = 232
Age [years], mean (SD)		10.10 (3.74)
Sex		
	Male, *n* (%)	129 (55.6)
	Female, *n* (%)	103 (55.6)
Health care system		
	Contributory, *n* (%)	188 (81.03)
	Subsidized, *n* (%)	43 (18.53)
	Private health care sector, *n* (%)	1 (0.43)
Weight °		
	Normal, *n* (%)	188 (81.03)
	Overweight or obesity, *n* (%)	42 (18.10)
Previous consultations		
	0, *n* (%)	40 (17.24)
	1, *n* (%)	146 (62.93)
	2, *n* (%)	38 (16.38)
	3, *n* (%)	8 (3.45)
Referred patient, *n* (%)		177 (76.29)
Symptoms		
	Vomiting, *n* (%)	210 (90.52)
	Anorexia, *n* (%)	184 (79.31)
	Nausea, *n* (%)	177 (76.29)
	Fever, *n* (%)	175 (75.43)
	Diarrhea, *n* (%)	97 (41.81)
High-risk patients, *n* (%)		75 (32.33)
PAS*		
	Low, *n* (%)	6 (2.59)
	Medium, *n* (%)	33 (14.22)
	High, *n* (%)	193 (83.19)
ASA+		
	I, *n* (%)	140 (60.34)
	II, *n* (%)	66 (28.45)
	III, *n* (%)	22 (9.48)
	IV, *n* (%)	4 (1.72)
Patients that had already been administered antibiotics, *n* (%)		9 (3.88)
Laboratory tests		
	Leukocyte counts [10^3/uL], median (p25-975)	16.11 (11.92–19.23)
	Neutrophil counts [10^3/uL], median (p25-975)	13.67 (9.92–17.06)
	CRP [mg/L], median (p25-975)	154 (105–307)
Ultrasound *n* = 99		
	Appendicitis, *n* (%)	41 (17.67)
	Suggestive changes, *n* (%)	31 (13.36)
	Normal, *n* (%)	27 (11.64)
Computed tomography *n* = 29		
	Appendicitis, *n* (%)	23 (9.91)
	Suggestive changes, *n* (%)	5 (2.16)
	Normal, *n* (%)	1 (0.43)

*SD* standard deviation, value over 230 patients, *PAS pediatric appendicitis score, ^+^*ASA* Physical Status Classification System of the American Society of Anesthesiologists, *CRP* C-reactive protein

Regarding laboratory testing, the median leucocyte count was 16,110, the median neutrophils count was 13,670, and the median C-reactive protein (CRP) value was 154. A pediatric appendicitis score (PAS) > 7 [23] was documented in 83.19% patients. Preoperative ultrasound was performed in 99 patients (42.67%), and appendicitis was diagnosed in 72.72% of them. Finally, computed tomography (CT) scan of the abdomen was necessary in 29 children, with a positive finding for appendicitis diagnosis in 96.55% of them (Table 1).

### Surgery

The median time between symptom onset and surgery was 52.57 h. 88.79% of the patients were classified as ASA I and ASA II by the anaesthesiologist according to the American Society of Anaesthesiologists physical status classification. In turn, 32.33% were classified as high-risk patients, and 9 of them had received an antibiotic prior to admission. Laparoscopic appendectomy was performed in 96.55% of the patients, and conversion to open surgery was required in 9 cases (3.88%). The surgical procedure was performed by a pediatric surgeon in 27.59% of the cases, while the remaining surgeries were performed by pediatric surgery residents assisted by a pediatric surgeon. The duration of the surgery (including the placement of the central access when needed) was 98.4 min on average. Localized peritonitis and generalized peritonitis were reported in 93 (40.08%) and 139 (59.92%) children, respectively. Postoperative central catheter placement was required in 130 patients (56.03%), and 135 patients (58.19%) required management in the intensive care unit (ICU) during the immediate postoperative period.

### Microbiology

Polymicrobial growth was obtained in 48 samples (20.69%) of the 232 positive cultures. *Escherichia coli *(*E*. *coli*) was the germ most frequently isolated (80.14%), followed by *Pseudomonas aeruginosa* (*P. aeruginosa*) (7.45%) and *Klebsiella pneumoniae* (2.83%). Among Gram-positive cocci, *Streptococcus anginosus* and *Streptococcus constellatus* were isolated, each, in 1.77% of samples (Table 2).

**Table 2:** Frequency of isolates of aerobic and anaerobic bacteria

Bacteria	No	%
Gram-negative		
*Escherichia coli*	226	80.14
*Pseudomonas aeruginosa*	21	7.45
*Klebsiella pneumoniae*	8	2.83
*Klebsiella oxytoca*	4	1.42
*Enterobacter aerogenes*	2	0.71
*Proteus mirabilis*	2	0.71
Gram-positive		
*Streptococcus anginosus*	5	1.77
*Streptococcus constellatus*	5	1.77
*Enterococcus avium*	1	0.35
*Enterococcus faecium*	1	0.35
Anaerobic		
*Prevotella disiens*	3	42.86
*Prevotella intermedia*	3	42.86
*Gemella morbillorum*	1	14.29

Regarding antimicrobial resistance profiles, 26.99% of *E. coli* isolates were found to be resistant to ampicillin/sulbactam, while only 0.44% were resistant to piperacillin/tazobactam. In addition, 5.31% of *E. coli* isolates were classified as extended-spectrum beta-lactamase (ESBL)-producing bacteria. No antibiotic resistance was reported in any *P. aeruginosa* isolate. For *K*. *pneumoniae* isolates, 25%, 12.5%, 12.5% and 12.5% were resistant to ampicillin/sulbactam, piperacillin/tazobactam, meropenem, and 3rd- and 4th-generation cephalosporins, respectively (Table 3).

**Table 3:** Antibiotic resistance profiles of the isolates

Microorganisms						
Antibiotics	n	Gram-negative			Gram-positive	
		*E. coli*	*P. aeruginosa*	*K. pneumoniae*	*S. anginosus*	*S. constellatus*
		226	21	8	5	5
ESBL*	%	5.31	–	0	–	–
Amp/sulba^&^	%R	26.99	–	25	–	–
Pip/Tazo^+^	%I	3.54	0	0	–	–
	%R	0.44	0	12.5	–	–
Meropenem	%R	0	0	12.5	–	–
Clindamycin	%R	–	–	–	0	0
Vancomycin	%R	–	–	–	0	0
Cefuroxime	%I	0	–	–	–	–
Ceftriaxone	%R	5.31	–	12.5	0	0
Cefepime	%R	5.31	0	12.5	–	–
Amikacin	%R	0	0	0	–	–
Ciprofloxacin	%I	5.31	0	0	–	–
	%R	10.62	0	12.5	–	–
Tigecycline	%R	0	–	0	0	0
Linezolid	%R	–	–	–	0	0
Penicillin G	%I	–	–	–	20	20

*ESBL extended-spectrum beta-lactamase, &Amp/sulba Ampicillin/sulbactam, + *Pip*/*tazo* piperacillin/tazobactam, *R* resistant, *I* intermediate susceptibility

A total of 50 cultures with germs resistant to the administered antibiotic were obtained. However, an antibiotic switch was required in only 5 cases (2.16%). In the remaining 45 (19.4%) cases, empirical antimicrobial therapy was not modified as the patients showed a good clinical evolution.

### Follow-up

During the postoperative period, 7 (3.01%) and 42 (18.10%) patients developed surgical wound infections and intra-abdominal abscesses, respectively, accounting for 21.11% of postoperative complications. Of the 42 children who developed intra-abdominal abscesses, 22 required drainages, which were performed by an interventional radiologist in 12 patients; 10 patients required re-interventions. Bacterial growth was obtained in 13 samples taken from intra-abdominal abscesses that were drained, for a total of 15 isolates. Within the antimicrobial resistance profile, a germ different from the one detected in the initial culture was documented in 2 patients, and antibiotic resistance mechanisms that were not identified in the initial culture were reported in 6 patients, as shown in Table 4.

**Table 4:** Culture isolates of the intra-abdominal abscesses

No	Initial culture	Antibiotic resistance profile	Culture of the intra-abdominal abscess	Antibiotic resistance profile	Was it the same germ? Yes/No	Susceptibility S/R
1	*Escherichia coli*	Natural	*Escherichia coli*	Natural	Yes	S
2	*Escherichia coli*	Natural	*Escherichia coli*	ESBL^&^	Yes	R
2	*Shigella sonnei*	AMPC^+^				
3	*Escherichia coli*	Natural	*Escherichia coli*	Penicillinase-Sensitive Inhibitors	Yes	R
4	*Enterobacter aerogenes*	AMPC^+^	*Enterobacter aerogenes*	AMPC^+^	Yes	R
4	*Streptococcus anginosus*	Natural	*Streptococcus anginosus*	Natural		
5	*Escherichia coli*	Natural	*Escherichia coli*	Natural	Yes	S
6	*Escherichia coli*	Natural	*Escherichia coli*	AMPC^+^	Yes	R
7	*Escherichia coli*	Natural	*Escherichia coli*	Natural	Yes	S
7	*Pseudomonas aeruginosa*	Natural				
8	*Pseudomonas aeruginosa*	Natural	*Pseudomonas aeruginosa*	Natural	Yes	S
8	*Escherichia coli*	ESBL^&^				
9	*Pseudomonas aeruginosa*	Natural	*Pseudomonas aeruginosa*	Natural	Yes	S
9	*Escherichia coli*	Penicillinase-sensitive inhibitors				
10	*Escherichia coli*	Penicillinase-sensitive inhibitors	*Escherichia coli*	Penicillinase-sensitive inhibitors	No	R
10			*Klebsiella oxytoca*	ESBL^&^		
11	*Escherichia coli*	Natural	*Escherichia coli*	Natural	Yes	S
12	*Escherichia coli*	Natural	*Escherichia coli*	Natural	Yes	S
13	*Escherichia coli*	Natural	*Streptococcus constellatus*	Modification of PBP	No	R

+AMPC AmpC-type beta-lactamases, ^&^*ESBL* extended-spectrum beta-lactamases, *S* susceptibility, *R* resistant

The average duration of antibiotic therapy was 8.55 days (SD 4.66). A total of 170 patients (73.27%) attended a postoperative follow-up visit within an average of 20.32 days (SD 8.20), and the other patients were contacted by telephone. Thirty-two patients were readmitted in the early postoperative period, and 15 of these readmissions (6.46%) were due to intra-abdominal abscesses or wound infection.

### Bivariate analysis

The development of intra-abdominal abscesses in the postoperative period was significantly associated with the following factors: longer time between symptom onset and surgery in patients with abscesses (66.5 h vs. 49.46 h, *p* < 0.01); being classified as a high-risk patient on admission [(52.38%) vs. (27.89%), *p* < 0.002]; being operated on by the pediatric surgeon [(40.48%) vs. (24.74%), *p* = 0.04]; longer duration of the surgery (105 min vs. 90 min *p* = 0.01); requiring conversion to open surgery (4 vs. 5, *p* = 0.04); presence of generalized peritonitis [(76.19%) vs (56.32%), *p* = 0.02]; postoperative central catheter placement [(78.57%) vs. (51.05%) *p* < 0.0001]; and requiring postoperative management at the ICU [(80.95%) vs. (53.16%) *p* < 0.0001] (Table 5). Symptoms, age, sex, PAS, and laboratory results were not statistically significant. Likewise, polymicrobial isolation was not statistically significant. However, it was found that initial treatment with piperacillin/tazobactam was associated with the development of intra-abdominal abscesses [(78.57%) vs (53.72%), *p* = 0.003].

**Table 5:** Bivariate analysis of the patients who developed with intra-abdominal abscesses

		Intra-abdominal abscess	Absence of intra-abdominal abscess	*P*-value
		Yes = 42, *n* (%)	No = 190, *n* (%)	
Time to surgery (hours)		66.50 (44.67–109.70)	49.46(34.81–74.49)	**0.01**
High risk				
	Yes	22 (52.38)	53 (27.89)	**0.00**
	No	20 (47.62)	137 (72.11)	
Operated by the pediatric surgeon or pediatric surgery resident				
	Surgeon	17 (40.48)	47 (24.74)	**0.04**
	Resident	25 (59.52)	143 (75.26)	
Duration of the surgery (minutes) [Median (p25–p75)]		105(90–124)	90 (65–120)	**0.01**
Conversion to open surgery				
	Yes	4 (9.52)	5 (2.63)	**0.04**
	No	38 (90.48)	185 (97.37)	
Central catheter requirement				
	Yes	33(78.57)	97(51.05)	**0.00**
	No	9 (21.43)	93 (48.95)	
ICU management				
	Yes	34 (80.95)	101 (53.16)	**0.00**
	No	8 (19.05)	89 (46.84)	
The antibiotic was developed to fight the isolated germ				
	Yes	37 (88.10)	145 (76.32)	**0.09**
	No	5 (11.9)	45 (23.68)	
Type of peritonitis				
	Generalized	32 (76.19)	107(56.32)	**0.02**
	Localized	10 (23.81)	83 (43.68)	

Bold indicates statistically significant *p* values

*PAS pediatric appendicitis score, +*ASA* Physical Status Classification System of the American Society of Anesthesiologists

The association between the use of an antibiotic developed to fight the isolated germ and the development of intra-abdominal abscesses was not statistically significant when confounder factors were controlled (OR 1.87; 95% CI: 0.66–5.33). On the contrary, being classified as a high-risk patient on admission (OR 2.89; 95% CI: 1.36–6.15) was independently associated with the development of intra-abdominal abscesses (Table 6).

**Table 6:** Multivariate analysis results

Collection	Odds ratio	[95% Confidence interval]		*p*-value
Previous use of antibiotic	1.87	0.66	5.33	0.24
Age	1.05	0.95	1.17	0.29
Laparotomy	1.12	0.21	5.83	0.89
High-risk patient on admission	2.89	1.36	6.15	**0.01**

Bold indicates statistically significant *p* values

## Discussion

The optimal management of acute appendicitis in children depends on both empirical antimicrobial therapy and early appendectomy, taking into account that it is recommended to administer the antibiotic within the first hour after making the diagnosis [11]. HOMI is a referral university hospital for the city of Bogotá and some neighboring departments, and it receives critically ill children (in fact, 76.29% of the patients included in the present study were referred). This explains the long median time elapsed between symptom onset and surgery (52.57 h), the high proportion of patients with generalized peritonitis (59.92%), and the high percentage of patients at high risk of presenting complications on admission (32.33%) observed in the present study.

Currently, there are multiple antimicrobial therapy regimes for patients with appendicitis, but the use of ampicillin-sulbactam is not recommended in developed countries given the high antimicrobial resistance rate to this antibiotic documented [12], which reaches 54.7% in the SMART study [13]. However, our study showed that the rate of resistance to ampicillin/sulbactam was lower (26.99%). At HOMI, ampicillin/sulbactam use is selected for the postoperative management of children without a history of hospitalization and those with a low risk and localized peritonitis (evidenced during the surgical procedure). In patients with generalized peritonitis or presenting with any high-risk factor, piperacillin/tazobactam administration is started postoperatively, as it has shown to be safe in the pediatric population [14, 15].

A systematic review of studies conducted only in children found that there was no significant difference in the development of surgical wound infections between children who received ampicillin/sulbactam and those who received cefotaxime + metronidazole [16]. Similarly, no difference was found in the clinical resolution of the infection between the use of ampicillin/sulbactam and the use of ertapenem [17]. In addition, it has been described that prolonging antibiotic treatment does not provide any clinical benefit in patients at high risk of treatment failure [18]. However, the present study found that there was an increased risk of intra-abdominal abscesses in patients who received piperacillin/tazobactam after surgery. This is inherently associated with its indication to treat high-risk patients with generalized peritonitis.

Antibiotic resistance is a growing problem worldwide. In this study, 5.31% of *E. coli* isolates were found to be ESBL-producing organisms, which is lower than the rate reported in the SMART study (6.8%) [13], but higher than the rates described in other studies [19]. This study also found that having a microorganism resistant to the antibiotic that the patient received was not significantly associated with the development of an intra-abdominal abscess in the postoperative period. Moreover, only 2.16% of patients required a switch in antibiotics, and this was mainly based on the patient’s clinical condition.

The medical literature describes many risk factors in children that are associated with the development of postoperative intra-abdominal abscesses, including the presence of diarrhea on hospital admission [20], obesity [21], prolonged duration of surgery [22, 23], the ASA physical status classification [22–24], intraoperative presence of fecaliths, and postoperative management in the ICU [25]. In this regard, the present study found that being classified as a high-risk patient on admission was independently associated with the development of intra-abdominal abscesses with an odds ratio of 2.89.

This study has several limitations. First, the sample size was small, which may have limited the ability to adjust for all possible confounding factors. Second, some possible confounders were not measured. Finally, the study was conducted at a referral hospital, which means that the patients included may have had different clinical characteristics than patients who are treated at other hospitals, thus possibly introducing selection bias into the results.

## Conclusions

In our study, *E. coli* was the most frequently isolated germ, with a low rate of ESBL-producing isolates. Having a microorganism resistant to the antibiotic used in the empirical therapy was not associated with an increased risk of postoperative abscess, while being a high-risk patient on admission, the presence of septic shock, previous use of antibiotics, and immunodeficiency are all associated with the development of intra-abdominal abscesses postoperatively. Based on these findings, it is recommended to optimize antibiotic therapy in this group of patients to reduce the risk of complications. However, it is important to note that these results are from a single-center study and need to be reproduced in a multi-center study to confirm the findings.
